# Phase III trial of postoperative cisplatin, interferon alpha-2b, and 5-FU combined with external radiation treatment versus 5-FU alone for patients with resected pancreatic adenocarcinoma – CapRI: study protocol [ISRCTN62866759]

**DOI:** 10.1186/1471-2407-5-37

**Published:** 2005-04-12

**Authors:** HP Knaebel, A Märten, J Schmidt, K Hoffmann, C Seiler, K Lindel, H Schmitz-Winnenthal, S Fritz, T Herrmann, H Goldschmidt, U Mansmann, J Debus, V Diehl, MW Büchler

**Affiliations:** 1 University of Heidelberg, Department of Surgery, Im Neuenheimer Feld 110,69120 Heidelberg, Germany; 2 University of Heidelberg, Department of Radiology, Im Neuenheimer Feld 400,69120 Heidelberg, Germany; 3 University of Heidelberg, Department of Medicine, Im Neuenheimer Feld 410,69120 Heidelberg, Germany; 4 LMU München, IBE Medical School, Marchioninistr. 15, 81377 München, Germany; 5 National Centre for Tumor Diseases, Im Neuenheimer Feld 350, 69120 Heidelberg, Germany

## Abstract

After surgical intervention with curative intention in specialised centres the five-year survival of patients with carcinoma of the exocrine pancreas is only 15%. The ESPAC-1 trial showed an increased five-year survival of 21% achieved with adjuvant chemotherapy. Investigators from the Virginia Mason Clinic have reported a 5-year survival rate of 55% in a phase II trial evaluating adjuvant chemotherapy, immunotherapy and external-beam radiation.

**Design:**

The CapRI study is an open, controlled, prospective, randomised multi-centre phase III trial. Patients in study arm A will be treated as outpatients with 5-Fluorouracil; Cisplatin and 3 million units Interferon alpha-2b for 5 1/2 weeks combined with external beam radiation. After chemo-radiation the patients receive continuous 5-FU infusions for two more cycles. Patients in study arm B will be treated as outpatients with intravenous bolus injections of folinic acid, followed by intravenous bolus injections of 5-FU given on 5 consecutive days every 28 days for 6 cycles. A total of 110 patients with specimen-proven R0 or R1 resected pancreatic adenocarcinoma will be enrolled. An interim analysis for patient safety reasons will be done one year after start of recruitment. Evaluation of the primary endpoint will be performed two years after the last patients' enrolment.

**Discussion:**

The aim of this study is to evaluate the overall survival period attained by chemo-radiotherapy including interferon alpha 2b administration with adjuvant chemotherapy. The influence of interferon alpha on the effectiveness of the patients' chemoradiation regimen, the toxicity, the disease-free interval and the quality of life are analysed. Different factors are tested in terms of their potential role as predictive markers.

## Background

Only 10–20% of patients with pancreatic cancer can be resected with curative intent at the time of diagnosis. Loco-regional recurrence and/or metastatic disease develop in the majority of patients who undergo pancreatic resection. Relapse occurs within 9–15 months after initial presentation and patients have median life expectancies of only 12–15 months without adjuvant therapy. The 5-year survival rate of patients with resected pancreatic adenocarinoma is approximately 20% [1]. The statistics for the 80 to 90 % of patients who present with locally advanced and metastatic pancreatic cancer are even more dismal. Rarely do such patients achieve a complete response to treatment; median survival is 5–10 months and 5-year survival is near zero [2].

There is very little randomised data on adjuvant therapy for pancreatic carcinoma. The ESPAC-1 trial assessed the role of adjuvant therapy in a randomized study. 548 patients were enrolled in Europe in this first large randomized trial. There was evidence that adjuvant chemotherapy brought survival benefits. The five-year survival rate was 21 percent among patients who received chemotherapy [3].

In 1995 a phase II trial was initiated by the Virginia Mason Clinic, Seattle, USA combining pancreaticoduodenectomy and adjuvant therapy with 5-FU, cisplatin, interferon alpha and radiation therapy. Picozzi et al. reported treatment and results in a series of 43 patients with high-risk resected pancreatic adenocarcinoma (84% node positive, 19% margin positive). After a median follow-up period of 32 months the 2-year survival rate was 64% and the 5-year survival rate was 55% [4]. The overall recurrence rate was 12% of which 80% occurred within 2 years after surgery [5].

Since the Virginia Mason group reported such encouraging results in improving survival periods, there has been considerable interest in gaining increased experience with this therapy combination in an effort to confirm results and evaluate the toxicity profile of a modified version of this regimen.

Radio-sensitising properties of 5-FU (5-fluorouracil) and cisplatin are well known. The incorporation of interferon alpha-2b into a combined modality treatment program seems to offer a number of theoretical advantages. These include: 1) the radio-sensitisation effects of interferon alpha-2b and 5-FU perhaps synergistically [6]; 2) enhanced 5-FU based bio-availability; 3) a synergistic inhibition of pyrimidine metabolism with 5-FU; and 4) an independent immunomodulatory effect of interferon alpha-2b [7]. 5-FU and interferon alpha-2b have been used together to advantage in several cancer settings, but not as part of a combined modality program [8]. Cisplatin also has a radio-sensitising effect and shares similar properties of cytotoxic synergy with interferon alpha-2b and 5-FU in both experimental and clinical cancer systems [9]. In a previous study we were able to demonstrate in vitro that interferon alpha has direct inhibitory properties and that it reduces the enhanced proliferation rate and VEGF secretion after a single treatment with cisplatin [10]. The use of interferon alpha-2b, 5-FU and cisplatin together thus might represent a kind of "combination radiosensitizer" analogous to combination chemotherapy potentially useful in the treatment of pancreaticobiliary cancers, especially from the standpoint of local control.

## Design

### Trial organization

CapRI has been designed by the Department of Surgery, University of Heidelberg. The trial is carried out by the National Centre for Tumourdiseases (NCT). The NCT is a multidisciplinary clinical and basic research group as well as comprehensive cancer centre of the Medical School of the University of Heidelberg that has been founded to improve care and research for cancer patients. The trial is sponsored by the Manfred-Lautenschläger-Foundation. The sponsor is not involved in the database management and has no access to the randomisation code.

### Coordination

The trial is co-ordinated by the Surgical Department in cooperation with the National Centre for Tumourdiseases (NCT) at the University of Heidelberg. The Dept. of Surgery is responsible for overall trial management, trial registration (International Standard Randomised Controlled Trial Number (ISRCTN 62866759), ), database management, quality assurance including monitoring, reporting and for the scientific program of all trial related meetings.

### Investigators

Patients will be recruited by the Department of Surgery at the University of Heidelberg. Due to the multi-modal nature of the trial, all investigators are experienced oncologists from the fields of hematology/oncology, radiation oncology and general surgery at the University of Heidelberg co-operating in this trial.

### Adverse events committee

This committee consists of 3 independent physicians (medical oncologist, radiation oncologist and surgeon) and decides on the final diagnostic classification of critical clinical events. For all serious adverse events the documentation and relevant patient data are verified by the co-ordinating personnel before submitting the data to the Adverse Events Committee for diagnostic classification.

Analysis of safety related data is performed with respect to frequency of:

• Serious Adverse Events and Adverse Events stratified by body-system

• Adverse Events stratified by severity

• Adverse Events stratified by causality.

Patient toxicities will be assessed using the NCI Common Toxicity Criteria (CTC). Toxicity will be evaluated pretreatment, weekly during chemoradiation/ chemotherapy, prior to each course of infusional 5-FU and at follow-up. Unacceptable toxicity is defined as unpredictable, or irreversible Grade 4 toxicity. Decisions regarding weekly chemoradiation treatment and chemotherapy dose-adjustment will be made using the guidelines below and based on hematological parameters (ANC and platelets) monitored weekly during chemoradiation before each dose of cisplatin.

### Medication supply

All chemotherapeutic and immunotherapeutic agents are prepared and provided by the pharmacy of the University Hospital Heidelberg. Medication will be prepared for each patient specifically and delivered just prior to administration to the NCT.

### On-site monitoring

During recruitment of patients monitoring on site is performed according to good clinical practice (GCP) guidelines. The monitoring for this trial will be performed by a monitor from a Clinical Research Organisation (CRO) who is not involved in the trial or in completion of the case report form (CRF). The data management will be performed by an independent medical documentalist from the Institute for Medical Bioinformatics, University of Heidelberg. The medical monitoring will be done by two independent oncologists not involved in conducting this trial.

### Ethics, informed consent and safety

The final protocol was approved by the ethics committee of the University of Heidelberg, Medical School (L-042/2003). This study complies with the Helsinki Declaration in its recent German version, the Medical Association's professional code of conduct, the principles of Good Clinical Practice (GCP) guidelines and the Federal Data Protection Act. The trial will also be carried out in keeping with local legal and regulatory requirements. The medical secrecy and the Federal Data Protection Act will be followed.

Written informed consent is obtained from each patient in oral and written form before inclusion in the trial and the nature, scope, and possible consequences of the trial have been explained by a physician. The investigator will not undertake any measures specifically required only for the clinical trial until valid consent has been obtained.

### Patient selection

CapRI focuses on hospitalised patients over 18 years of age treated with pancreatic head resection for pancreatic adenocarcinoma during an 18-month period started in August 2004. Men and women over eighteen years of age with biopsy-proven completely resected (R0 or R1) pancreatic adenocarcinoma of the pancreatic head or uncinate process will be screened for participation in the study. A detailed overview of all eligibility criteria is given in Table 2.

### Study design

The CapRI study is designed as an open, controlled, prospective, randomised potentially multi-centre trial meant to evaluate the post-operative overall survival of patients with pancreatic adenocarcinoma receiving chemo-radiotherapy including interferon alpha 2b administration compared with adjuvant chemotherapy. The treatment is offered to a heterogeneous group of people under clinical circumstances, covering a wide age range, for both sexes and with heterogeneous characteristics / co-morbidities.

One year after inclusion of the first patient an interim analysis will be performed. If at this time point there is already a significant difference between both groups favouring the Virginia Mason scheme (p < 0.001) group B (5-FU/Folinic acid) will be stopped and replaced by group C (chemoradiation without interferon alpha). This group will help to define the possibly deciding influence of interferon alpha on the effectiveness of the Virginia Mason treatment protocol. Addition of group C to the study would be the matter of a separate study amendment. The study design will not be changed prior to agreement of the ethics committee.

### Study objectives

The primary objective is to compare the overall survival at two years postoperatively between two different methods of adjuvant treatment: therapy with 5-FU, cisplatin, interferon alpha 2b combined with radiation and the standard treatment from the ESPAC-1-Trial with 5-FU plus folinic acid.

Secondary objectives are to determine the role and the mechanism of interferon alpha 2b in patient's chemoradiation regimen, the toxicity, the disease-free interval and the quality of life. Different factors are tested in terms of their potential role as predictive markers.

### Randomisation and standardised treatment scheme

A block-randomisation-list is generated via computer system (SAS Version 8.2, SAS Institute Inc., Cary, USA). The sealed randomisation list is stored in the investigator file. Patients are randomised using sealed opaque envelopes in the independent study centre at the Department of Surgery (Clinical Study Centre – "Klinisches Studienzentrum Chirurgie" – KSC) until informed consent is attained and diagnostic procedures rule out any contra-indication for participation in this trial.

After randomisation and pre-treatment evaluation treatment must begin within 12 weeks of surgery. A port catheter will be placed after informed consent and randomisation will be done within one to two weeks after pancreatoduodenectomy for patients in study arm A. These patients will be treated with 200 mg/m^2^/day 5-FU by continuous intravenous infusion at days 1–38.

Cisplatin – 30 mg/m^2 ^(maximum single cisplatin dose of 60 mg) iv over 60 minutes on days 1, 8, 15, 22, 29, 36 (6 doses). Two to three hours before and after cisplatin administration the patients will receive hydration of at least 2 litres.

Interferon alpha-2b (Intron A) will be administered at a dose of 3 million units subcutaneously three times weekly (Monday, Wednesday, and Friday) for 17 doses. Non-steroidal anti-inflammatory drugs and steroids should be avoided if possible during Intron A treatment.

External beam radiation is to be given concurrently with chemotherapy with a total dose of 50.4 Gy in 28 fractions over 5.5 weeks (1.8 Gy/day). The pancreatic bed (i. e. resection margin) will be covered with a minimum margin of 2 cm. The hepatoduodenal ligament, origins of the celiac axis and superior mesenteric artery will be included. The AP/PA fields must include the entire duodenal C-loop as seen on the pre-operative CT scan. All patients will receive XRT per informed consent. A four-field technique with AP/PA and lateral fields and customised blocking is required. The beams will be weighted more heavily in the AP/PA fields (usually 2:1 compared to the lateral fields). The dose contribution from the lateral fields should be restricted to 20 Gy. Simulation should be done with the patient in the supine position with "arms up" position. A dosage greater than or equal to 10 MV photons should be used.

In study arm A the patients receive post-chemoradiation 5-FU infusions of 200/mg/m^2 ^/day by continuous intravenous infusion on days 64–101 and 120–161. The timing of these courses of infusional 5-FU will be adjusted in patients who have treatment interruptions.

Patients in study arm B will be treated with 20 mg/m^2 ^intravenous bolus injection of Folinic acid, D-L form, followed by 425 mg/m^2^/day intravenous bolus injection of 5-FU given on 5 consecutive days every 28 days for 6 cycles, i.e. 24 weeks.

**Figure 1:**
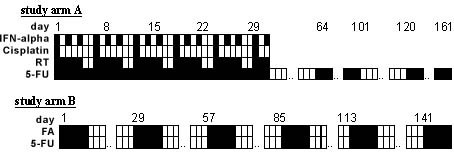
CapRI treatment scheme.

The treatment protocol is outlined in figure 1.

### Investigation schedule and follow-up

Pre-treatment evaluation for patients who are enrolled in study arm A includes a single low-dose (3 Mio U) injection of INTRON A prior to therapy (LDI). Blood will be drawn for intensive immunological studies. All patients (study arm A or B) must have appropriate lab and radiographic studies (CXR; CT abdomen [done post-operatively]; CBC; platelet count; BUN; creatinine; bilirubin, CA 19-9, and CEA) conducted prior to study enrolment to meet eligibility criteria.

During days 1–38 [study arm A] or during chemotherapy [study arm B] patients will be assessed with laboratory evaluation: complete blood count and blood chemistries weekly. Laboratory parameters in study arm A will be evaluated before each dose of cisplatin. Creatinine will be determined also one day after administration of cisplatin. Blood from members of study arm A will be investigated weekly for immunological markers.

Vital signs (blood pressure and pulse rate) and temperature are controlled daily during treatment. Patients are evaluated prior to receiving chemoradiation or chemotherapy. Patients enrolled in study arm A are evaluated weekly by the medical oncology and radiation oncology team during treatment. The team will check patients at each visit for symptoms due to therapy; a physical examination and complete safety labs should be performed. The patients' mental state will be investigated weekly. The questionnaire CES-D will be used as support. The three module questionnaire will be filled out during weeks 3 and 6 (study arm A) or week 3 and week 24 for study arm B.

During post-chemoradiation, infusional 5-FU (Study Arm A) patients will be evaluated by a physician prior to treatment and every 2 to 3 weeks with clinical assessment and laboratory parameters including a CBC, electrolytes, BUN, and creatinine. If patients undergo post chemoradiotherapy infusional 5-FU coordinated by an outside medical oncologist, a study nurse will contact the patient at least once weekly by telephone.

In the post-treatment period patients will be seen every 3 months by the surgical service for the first 2 years, every 4 months for the third year, and every 6 months during the 4th and 5th post-treatment years.

The aggregate clinical, laboratory, and imaging evaluations required per protocol as well as the timing of the optional three module questionnaire are outlined in table 1.

The follow-up will be continued for two years. Follow-up data of overall survival will be evaluated annually.

### Assessment of quality of life

Measurement of quality of life is one of the secondary objectives of the trial. Overall survival, return to previous employment as well as persistence of symptoms, the ability to perform appropriate activities and to care for oneself are criteria applied in the three questionnaires used in this study.

EORTC QLQ-C30 is a general measure of qualitiy of life in cancer patients. It incorporates nine multi-item scales: five functional scales (physical, role, cognitive, emotional, and social); three symptom scales (fatigue, pain, and nausea and vomiting); and a global health and quality-of-life scale [17]. Specific symptoms (dyspnoea, insomnia, anorexia, constipation, diarrhoea, and financial impact) are measured as six single items. This instrument has been used extensively with a variety of cancer patients and was able to discriminate between individuals with metastatic and non-metastatic disease, as well as between patients at different stages of illness. The scale has good internal consistency (alpha > 0.70), and good test re-test reliability (0.80 to 0.90) [18].

To assess disease-specific symptoms for patients with pancreatic cancer the pancreatic specific module (QLQ-PAN26) [19] that has been designed to use long with the general measure is used in this study.

The CES-D [20] is a 20-item self-report measure of depression that emphasises the emotional dimension. Its emphasis on the affective components of depression and is preferable for use in medical populations. It has demonstrated high internal consistency in both the general population and in patient populations and convergent validity with other measures of depression [21].

### Evaluation of the role of interferon alpha

In addition to the Virginia Mason and the MD Anderson study, an investigation of the effects and mechanism of INTRON A will be performed. IFN alpha seems to have the greatest activity of all interferons in malignancies [21-23] but its mode of action is poorly understood.

It probably consists of a combination of stimulation of cell-mediated cytotoxicity [24,25], direct antiproliferative anti-tumour activity, and an anti-angiogenic effect [15,26-31]. The known molecular and cellular effects of IFN-alpha appear to complement the mechanism of action of other therapies [28].

In view of the significant improvement in survival rates achieved by using interferon alpha in the Virginia Mason study, studies are now needed to define the molecular and immunologic mechanism(s) of this modality. If the mechanism of action of IFN-alpha is more clearly understood it can be applied more selectively and its therapeutic index will be enhanced.

Patients in study arm A at the CapRI trial will receive a single low-dose injection of 1 million units (LDI) prior to therapy. Accompanying immunological analysis should help to define predictive response markers. During therapy patients blood samples of approx. 20 ml will be drawn once a week for immunological studies. The panel will include immunophenotypings, detection of cytokine mRNA in lymphocytes, determination of cytokine patterns in peripheral blood, cellular cytotoxicity assays against autologous tumor cells and determination of apoptotic effects of interferon against autologous tumor cells.

### Statistical considerations and sample size estimation

The primary endpoint in this study is the overall survival period, measured from the date of resection. The sample size calculation is based on the assumption of a constant monthly hazard rate of 0.044 in the standard group and a constant monthly hazard rate of 0.021 in the experimental group. The hazard values are derived from two-year survival rates in the study groups (using the formula λ_group _= -1 × ln(P_24,group_)/24) with a two-year survival rate of 35% in the control group [11,12]and a conservative two-year survival rate of 60% in study arm A[13].

Assuming an accrual period of 18 months and a follow-up of 42 months [14,15], testing for a difference in hazard (hazard ratio ≠ 1) on level α = 0.05 and with a power of 80% a study sample size of 96 patients is needed. Taking into consideration the estimate of approximately 14 patients which will not complete the treatment, a total number of 110 patients should be randomised.

The overall survival will be summarised by Kaplan-Meier estimate and differences in therapy protocols will be analysed by univariate Cox-regression.

Various secondary endpoints will be evaluated in this study as well. Disease free survival, measured from date of operation, will be summarised by Kaplan-Meier estimate.

One year after inclusion of the first patient an interim analysis will be carried out which could result in stopping arm B. The planning of the study is based on a fix sample approach and not on groups sequential. Because the alpha level of the interim analysis is set to α = 0.001, the sample size calculated from a group sequential approach will be similar to the sample size of the fixed study approach.

The main evaluation will be performed two years after the last patient's enrolment.

There will be explicit stopping rules in place to terminate the trial early in the unlikely event that an unacceptably high rate of treatment related deaths (TRD) is observed. TRD will be monitored using the design of Thall and Simon [16]. A non-informative Beta prior distribution (i.e., B (0.015, 0.085) for TRD rate is assumed. The trial will be stopped if at any point during the trial there is a greater than 90% probability that the true TRD rate is greater than 0.05. Each patient will subsequently be evaluated and, an independent safety board will be consulted in making decision.

In view of the poor prognosis of the patient group, there will be no explicit stopping rules based on the overall number of toxicities, since even high rates of reversible toxicities seem acceptable if there is a large survival gain. Patients can withdraw from study participation at any time. Patients are taken off the study if unacceptable toxicity appears. Unacceptable toxicity is defined as unexpected serious side effects or irreversible Grade 4 toxicity. Patients who withdraw from the study may be treated with 5-FU and folinic acid or with gemcitabine. The decision will be based on the individual reasons for withdrawing from the study.

One year after inclusion of the first patient an interim analysis will be done. If at this time point there already is a significant difference between both groups favouring the Virginia Mason scheme (p < 0.001) group B (5-FU/Folinic acid) will be stopped and replaced by group C (chemoradiation without interferon alpha).

## Discussion

The role of adjuvant therapy in potentially curatively resected adenocarcinoma of the pancreas remains a matter of debate. As the first, large multi-centre randomised controlled trial (RCT), the ESPAC-1-Trial clearly favoured adjuvant chemotherapy over postoperative chemoradiation [3]. However the quality of the radio-chemotherapeutic regimen in the ESPAC-1-Trial has been disputed. The Virgina Mason study group in Seattle, USA, published very promising data in phase-II-study involving immunotherapy and chemoradiation in the adjuvant setting [4]. The reliability of the data has been intensively discussed and there even a source-data-verification was performed by the National Cancer Institute (NCI). In the current setting with a reference adjuvant treatment from an RCT and very promising data from a phase-II-trial, there is the ideal basis for a controlled trial comparing the two most current and successful regimen. Being a centre focusing on pancreatic diseases and especially malignancies we therefore planned and conduct such a trial.

The CapRI study is an open, randomised controlled trial investigating the survival of patients after potentially curative resection of a pancreatic adenocarcinoma treated adjuvant with 5-FU, cisplatin, INTRON A combined with radiation or 5-FU plus folinic acid. The role and the mechanism of interferon alpha 2b in patient's chemoradiation regimen are evaluated. The toxicity, the disease-free interval and the quality of life are assessed. Different factors are tested for a potential role as predictive marker.

The results of the CapRI trial will definitely advance clinical and scientific knowledge on the adjuvant treatment of pancreatic carcinoma as it may confirm or de-mystify the remarkable results from the Virginia Mason study group.

## Abbreviations

CCC Comprehensive Cancer Centre

CRF Case Report Form

CRO Clinical Research Organisation

GCP Good Clinical Practice

ICH International Conference on Harmonisation of Technical

Requirements for Registration of Pharmaceuticals for

Human Use

IEC Independent Ethics Committee

ITT Intention-to-treat-analysis

NCT National Centre for Tumourdiseases

RCT Randomised Controlled Trial

SDGC Study Centre of the German Surgical Society

## Competing interests

The author(s) declare that they have no competing interests.

## Authors' contributions

The first three authors contributed equally. AM, JS, MWB planned, coordinated and conducted the study. Medical care is covered by KL, KH, HSW, SF, HT and TH. HPK and CS recruited patients and provided randomization. HG, VD and DJ took part in conducting the study. Scientific program is planned by AM and carried out by AM and KH. All authors read and approved the final manuscript.

## Note

Table 1: Investigation schedule

Table 2: Eligibility Criteria

## Pre-publication history

The pre-publication history for this paper can be accessed here:
